# Inhibitors of dermatan sulfate epimerase 1 decreased accumulation of glycosaminoglycans in mucopolysaccharidosis type I fibroblasts

**DOI:** 10.1093/glycob/cwae025

**Published:** 2024-05-17

**Authors:** Marco Maccarana, Binjie Li, Honglian Li, Jianping Fang, Mingjia Yu, Jin-ping Li

**Affiliations:** Department of Medical Biochemistry and Microbiology, University of Uppsala, Husargatan 3, 75123, Uppsala, Sweden; Beijing Advanced Innovation Centre for Soft Matter Science and Engineering, Beijing University of Chemical Technology, Beijing, No. 15 North Third Ring Road East, Chaoyang District, Beijing, 100029, P. R. China; Department of Medical Biochemistry and Microbiology, University of Uppsala, Husargatan 3, 75123, Uppsala, Sweden; GlycoNovo Technologies Co. Ltd., Room 202, Building 83-84, 887 Zuchongzhi Road, Pilot Free Trade Zone, Shanghai 201203, China; School of Chemistry and Chemical Engineering, Beijing Institute of Technology, No 8 and 9 Yards, Liangxiang East Road, Fangshan District, Beijing 102488, China; Department of Medical Biochemistry and Microbiology, University of Uppsala, Husargatan 3, 75123, Uppsala, Sweden; SciLifeLab, Uppsala University, Husargatan 3, 75123, Uppsala, Sweden

**Keywords:** DS-epi1, inhibitors, MPS-I, substrate reduction therapy

## Abstract

Genetic deficiency of alpha-L-iduronidase causes mucopolysaccharidosis type I (MPS-I) disease, due to accumulation of glycosaminoglycans (GAGs) including chondroitin/dermatan sulfate (CS/DS) and heparan sulfate (HS) in cells. Currently, patients are treated by infusion of recombinant iduronidase or by hematopoietic stem cell transplantation. An alternative approach is to reduce the L-iduronidase substrate, through limiting the biosynthesis of iduronic acid. Our earlier study demonstrated that ebselen attenuated GAGs accumulation in MPS-I cells, through inhibiting iduronic acid producing enzymes. However, ebselen has multiple pharmacological effects, which prevents its application for MPS-I. Thus, we continued the study by looking for novel inhibitors of dermatan sulfate epimerase 1 (DS-epi1), the main responsible enzyme for production of iduronic acid in CS/DS chains. Based on virtual screening of chemicals towards chondroitinase AC, we constructed a library with 1,064 compounds that were tested for DS-epi1 inhibition. Seventeen compounds were identified to be able to inhibit 27%–86% of DS-epi1 activity at 10 μM. Two compounds were selected for further investigation based on the structure properties. The results show that both inhibitors had a comparable level in inhibition of DS-epi1while they had negligible effect on HS epimerase. The two inhibitors were able to reduce iduronic acid biosynthesis in CS/DS and GAG accumulation in WT and MPS-I fibroblasts. Docking of the inhibitors into DS-epi1 structure shows high affinity binding of both compounds to the active site. The collected data indicate that these hit compounds may be further elaborated to a potential lead drug used for attenuation of GAGs accumulation in MPS-I patients.

## Introduction

Alpha-L-iduronidase (*IDUA*) is the only enzyme responsible for catabolism of iduronic acid (IdoA) in chondroitin/dermatan sulfate (CS/DS) and heparan sulfate (HS) in the lysosomes (Hampe et al. 2020). Deficiency in the gene encoding for this enzyme leads to accumulation of CS/DS and HS in cells, resulting in distorted cellular functions in many organs and causing disease denoted as mucopolysaccharidosis type I (MPS-I). MPS-I is divided into three clinical syndromes with decreasing clinical severity: Hurler syndrome (MPS I-H), Hurler-Scheie syndrome (MPS I-H/S), and Scheie syndrome (MPS I-S) (Clarke 2008). Although MPS-I is a rare disease, the genetic defect affects children, bringing huge family and social burden.

The CS/DS and HS are sulfated glycosaminoglycans (GAGs), expressed in all mammalian cells, having essential biological functions. This group of GAGs (referred in this report) are synthesized in the Golgi in the forms of proteoglycan and transported to cell surface and ECM for the diverse biological functions. For metabolism, the polysaccharides are internalized into the cellular lysosome where they are degraded sequentially by different exo-glycosidases and sulfatases. These enzymes are extremely substrate-specific and there is no redundant gene as compensation. Therefore, lacking any of the enzymes will result in a “blockage” of the CS/DS and HS catabolism, leading to lysosomal storage diseases (LSDs) including MPS-I. This storage affects a broad spectrum of molecular activities in several cellular pathways (Gaffke et al. 2021).

In CS/DS biosynthesis the glucuronic acids in the polymers are converted to its epimer IdoA catalyzed by dermatan sulfate epimerase 1 and 2 (DS-epi1 and DS-epi2) (Malmstrom et al. 2012). DS-epi1 and 2 are ubiquitously expressed but DS-epi1 is responsible for the majority of IdoA biosynthesis in all organs except brain (Maccarana et al. 2006). Elimination of DS-epi1 resulted in perinatal death and developmental defects of newborn mice of pure C57BL/6 or NFR genetic background, while mice with a mixed C57BL6/Sv129 genetic background were vital and had a normal life span. The only obvious phenotype was altered skin collagen fibers, causing skin fragility (Maccarana et al. 2009). Whereas the DS-epi1 heterozygous mice produced approx. 50% of the enzyme and did not show any phenotype, indicating that reduction of 50% of DS-epi1 had no physiological impairment in mice. In comparison, elimination of DS-epi2 did not lead to an overt phenotype, indicating that DS-epi1 is the major contributor for production of IdoA.

Currently, the clinical status and prognosis of MPS-I patients are helped by either hematopoietic stem cell transplantation or iduronidase enzyme replacement (ERT). These treatments have several disadvantages including limited effects in some organs, e.g. brain and cardiovascular system (Chen et al. 2019). Moreover, the active enzyme needs to be infused, with the potential risk of immune responses. With the high cost of enzyme production and transplantation procedure, these strategies have been exclusively limited to wealthy community. Therefore, to develop cheaper and easy-to-use medicines is desirable for all MPS patients. Our previous study showed that a drug, ebselen (2-phenyl-1,2-benzoisoselenazol-3(2H)-one), can efficiently inhibit enzymatic activity of IdoA producing enzymes and significantly reduced accumulation of the GAGs in the lysosome of fibroblasts obtained from MPS-I patient (Maccarana et al. 2021). However, ebselen failed to show effects in a MPS-I mouse model, probably due to its multiple targets of diverse biological pathways, including anti-oxidation and anti-inflammation (Azad and Tomar 2014). Nevertheless, the study has approved the concept of substrate reduction therapy (SRT) for MPS-I as an alternative strategy.

In this continued study, we have identified small molecules that displayed specific inhibitory activity towards DS-epi1. Screening of a dedicated library composed of 1,064 compounds found 17 inhibitors with IC_50_ around 10 μM. Two of the inhibitors, structurally clustering in two different structural groups, were investigated in cellular models. The results show that both inhibitors were able to reduce IdoA production and the accumulation of GAGs in patient derived MPS-I fibroblasts. Docking of the compounds into DS-epi1 molecule revealed the binding in the catalytic site. In consideration of the fact that reduction of DS-epi1 in mice did not cause abnormal biological functions, the substrate-reduction therapy through inhibition of the enzyme may be adapted for treatment of MPS-I patients. Thus, the novel compounds reported here should warrant for further medicinal chemistry optimization and pharmaceutical development.

## Results

### Reduced GAG content in MPS-I fibroblasts lacking DS-epi1

**Fig. 1:**
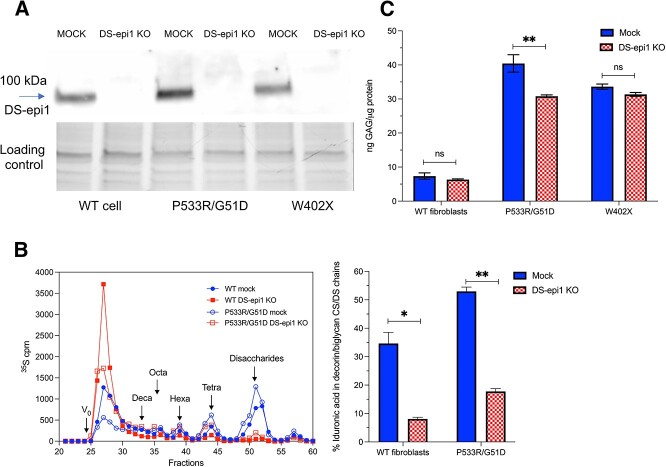
Decreased iduronic acid biosynthesis and GAG accumulation in DS-epi1eliminated fibroblasts. A) DS-epi1 was depleted by CRISPR-Cas9, as assessed by Western blot using anti-DS-epi1 antibody. The DS-epi1 eliminated cells are denoted as “KO” in the figure. The loading control shows the proteins blotted to the membrane (see “methods”). (B) GAGs in WT or P533R/G51D MPS-I fibroblasts, either mock or DS-epi1 KO, were metabolically labeled with ^35^S-sulfate for 24 h. Decorin/biglycan were isolated from medium. CS/DS chains were released by alkali treatment and digested by chondroitinase B. The digested products were analyzed on Superdex peptide (*left*). The iduronic acid content was quantified by calculation of the chondroitinase B resistant fragments (*right*). C) Unlabeled total GAGs isolated from the cells were measured by carbazole reaction and normalized to the total proteins in the cells. A and B) Similar data were obtained by triplicates analysis of samples from two cell clones.

Our earlier study showed that inhibition of DS-epi1 activity by ebselen reduced iduronic acid (IdoA) content of CS/DS in MPS-I fibroblasts and decreased pathological GAG burden in the cells (Maccarana et al. 2021). To further confirm the findings, we inactivated the *DSE* gene, coding for dermatan sulfate epimerase 1 (DS-epi1), by the CRISPR-Cas9 method in two MPS-I and one WT control fibroblasts. Screening of the transfected cells identified clones in which DS-epi1 was completely abolished, as assessed by Western blot (Fig. 1A). As controls, the cells were also mock-infected, which did not affect DS-epi1 expression. To confirm the effect of DS-epi1 abolishment on CS/DS structure, one MPS-I (P533R/G51D) and the WT fibroblasts were metabolically labeled with ^35^S-sulfate. After elimination of HS by deaminative cleavage, CS/DS proteoglycans in the culture medium were separated by size exclusion chromatography to obtain decorin and biglycan. The CS/DS chains were released from the core proteins by alkaline beta-elimination and degraded by chondroitinase B. Size exclusion chromatographic analysis of the degraded products shows the profile of chondroitinase B resistant oligosaccharides that contain internal glucuronic acid (GlcA) residues (Fig. 1B, left panel). The CS/DS from mock transfected cells (both WT and P533R/G51D) displayed a spectrum of chondroitinase B degraded oligosaccharides, indicating distribution of IdoA residues along the chains; in comparison, the CS/DS from DS-epi1 eliminated cells showed mainly one peak eluted at the void volume of the column, indicating lacking of IdoA in the chains. Calculation of radioactivity from the peaks found that the IdoA proportion in P533R/G51D cells is more than 50%, while only 35% in WT cells (Fig. 1B, right panel). Elimination of DS-epi1 resulted in significantly reduced IdoA in both cell types (from 35 to 8% in the WT cells and from 53 to 18% in the P533R/G51D cells). Quantification of the total GAG content shows a much higher level of total GAGs in both MPS-I fibroblasts than that in the WT cells (6–7 folds) (Fig. 1C). The total GAGs were slightly reduced in the DS-epi1 eliminated WT cells and W402X, but substantially reduced in the DS-epi1 eliminated P533R/G51D MPS-I cells, from 40 ng/μg protein to 34 ng/μg protein.

### Identification of DS-epi1 inhibitors

**Fig. 2:**
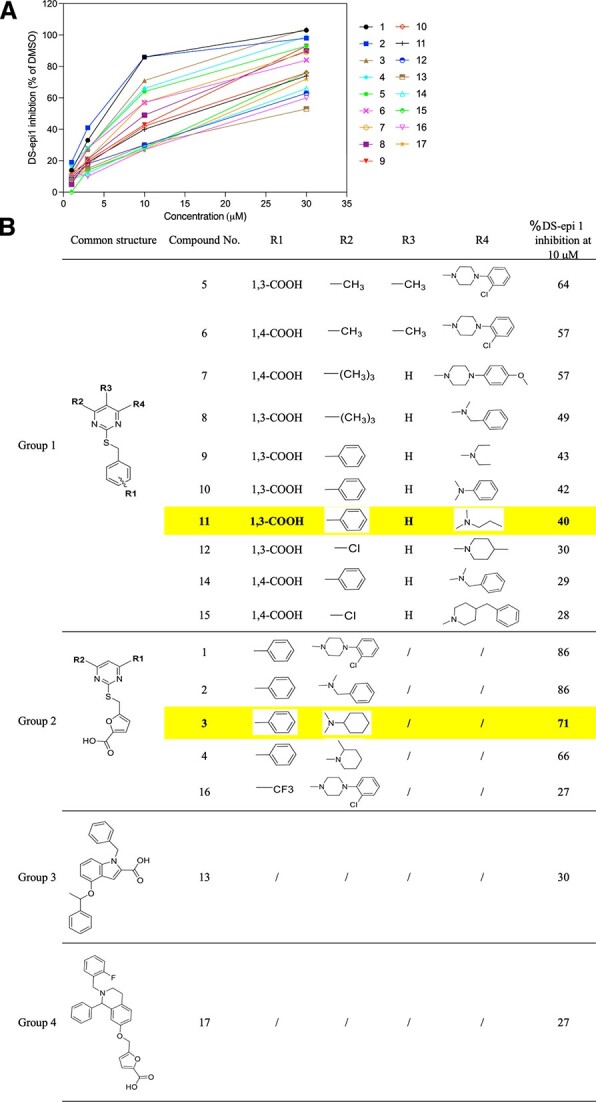
Identification of inhibitors. A) The chemical library of 1,064 compounds were screened for inhibition of DS-epi1 activity using recombinantly produced enzyme. The top scored 17 compounds are shown. B) The inhibitors are sub-grouped by chemical structures and their inhibitory potency at 10 μM is shown.

Having seen that elimination of DS-epi1 has resulted in reduced IdoA content and GAGs in the patient cells, it is rationalized that inhibition of DS-epi1 activity may help to reduce GAG accumulation in the MPS-I cells. To look for chemical compounds that can specifically inhibit DS-epi1 activity, we have used the crystal structure of chondroitinase AC for virtual screening a chemical library, because the DS-epi1 crystal structure was not available at that time, while chondroitinase AC shares similarities with DS-epi1 in recognition of glucuronic acid as substrate (Pacheco et al. 2009a). Based on the virtual screening results, a chemical library of 1,064 compounds was established and used for evaluation of DS-epi1 inhibitory activity. From the initial screening, we have found 17 compounds that displayed varied degree of inhibitory activity, between 27 to 86% at the concentration of 10 μM (Fig. 2A and B). One common feature of these compounds, which are structurally clustered into four groups, is the presence of one carboxyl group. This most likely mimics the substrate of DS-epi1 that converts the carboxyl group in the hexuronic acids. Restricted by the micro-amount of the chemicals in the library, one compound from each of the two major groups was selected for further investigation. These two compounds were resynthesized (Supplementary info) in sufficient chemical amount for the following studies, denoted as inhibitor **3** and **11**.

### Validation of the selected inhibitors

**Fig. 3:**
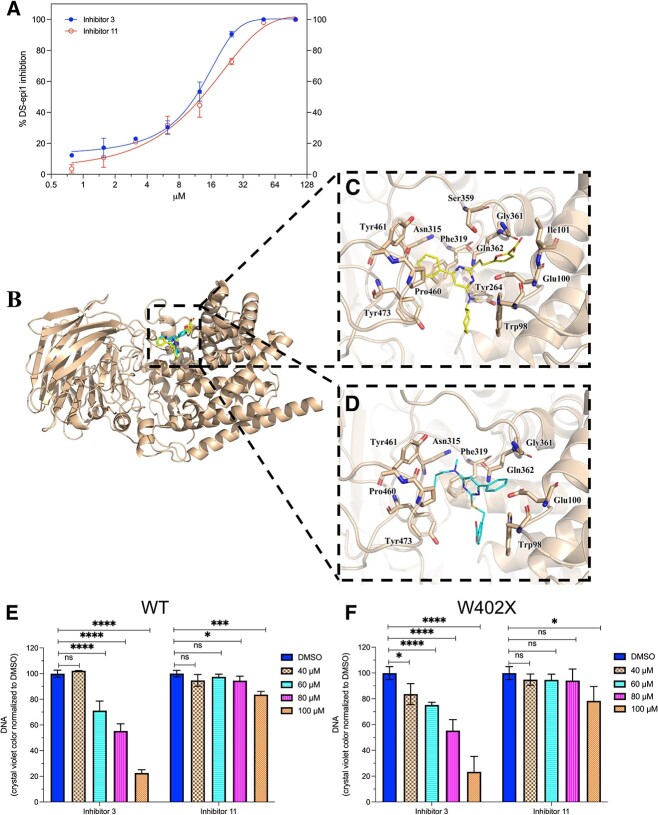
Characterization of selected inhibitors **3** and **11**. A) Inhibition curves in DS-epi1 enzymatic assay. Each single point represents value of quadruplicates and the experiment has been repeated three times. B) Computer-aided analysis shows that both inhibitors were docked into the active site of curved cleft of DS-epi1. Highlight of the amino acids that interact with the inhibitor **3** (C) and **11** (D) through hydrophobic contacts. E and F) Toxicity assessment of the inhibitors. WT (E) and W402X (F) fibroblasts were cultured in the 96-well plates in the presence of the inhibitors at the concentrations indicated. After six days of cultivation, DNA content of the cells was measured by crystal violet staining. The experiments in E and F was repeated three time and each point was in quadruplicates.

Analysis of the inhibitory activity towards recombinant DS-epi1 showed that **3** and **11** had a similar IC_50_, 12 μM and 16 μM, respectively (Fig. 3A). To get some insights for the inhibitory mechanisms, the interaction of the two inhibitors with DS-epi1 was investigated by computer aided study. Molecular docking of the inhibitors into DS-epi1crystal structure revealed that both compounds bind to DS-epi1, docking into the active site of the curved cleft (Fig. 3B–D) (Pacheco et al. 2009a; ). Detailed analysis shows the binding between the inhibitors and DS-epi1 is mainly through hydrophobic interactions, involving 12 amino acids for inhibitor **3**, and 9 amino acids for inhibitor **11**. In agreement with the activity assay, in silico binding studies showed that inhibitor **3** binds more tightly than inhibitor **11** with human DS-epi1 (Supplementary Table 1). In silico, inhibitor **3** bound more tightly than inhibitor **11** also with chondroitinase AC, which is the protein used for virtual screening to obtained the chemical library (Supplementary Table 2). In addition, the results indicate that inhibitor **3** and **11** have very similar affinities when the binding to DS-epi1 and chondroitinase AC was compared.

Before evaluation of the functional effect in cells, the potential cytotoxicity of the inhibitors was assessed. Viability of both WT and W402X fibroblasts was not affected after culturing for 48 h in the presence of the inhibitors at the concentration of 40–100 μM, as determined using resazurin-based reagent (data not shown). However, inhibitor **3** exhibited growth inhibitory effect after extended (6 days) cultivation of both cells, at the concentration of ≥60 μM (Fig. 3E and F). Therefore, inhibitor **3** in the following assays was limited to 50 μM. Nevertheless, inhibitor **11** did not affect the cell growth at the concentration as high as 80 μM and showed marginal toxicity at 100 μM. Thus, 80–100 μM were used in the following experiments.

### Reduced GAG accumulation in MPS-I fibroblasts treated with the DS-epi1 inhibitors

**Fig. 4:**
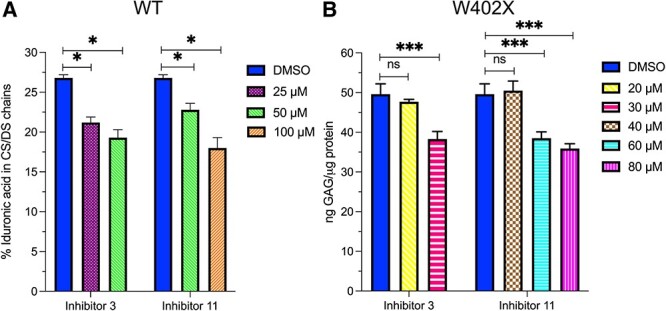
Effects of inhibitors **3** and **11** on fibroblasts. A) Reduced levels of IdoA in WT fibroblasts after treatment with inhibitors. The GAGs were metabolically labeled with ^35^S-sulfate for 24 h in the presence of inhibitor **3** and **11**. Total labeled CS/DS chains were recovered from medium and quantification of iduronic acid was performed as described in Fig. 1. B) MPS-I W402X fibroblast were treated for 10 days with the inhibitors, and the medium was changed every second day with fresh inhibitors. The total GAGs were isolated from the cells and measured by carbazole reaction. The experiments were repeated twice and the assays were in triplicates.

For assessment of the functional effect of the inhibitors, WT cells were treated with both inhibitors and the consequence on IdoA production was evaluated. The CS/DS chains from cell culture medium were isolated from the treated and untreated cells, cleaved by chondroitinase B, and the size analysis of the degraded products allowed quantification of IdoA, as described above. Inhibitor **3** reduced IdoA from 27% to 21 and 19% of CS/DS, at the concentration of 25 and 50 μM, respectively (Fig. 4A). While treatment with **11** led to a reduction of IdoA to 23% at the concentration of 50 μM, and to 18% at the concentration of 100 μM. Thus, inhibitor **3** seems having higher potency than **11** in the cells, which is agreed with the in vitro inhibitory activity. Further, we examined the effect of the inhibitors on total GAG production in W402X cells. Quantification of the total GAGs isolated from the cells treated with the inhibitors for 10 days shows a significant reduction of the GAG level, from 50 ng GAG/μg protein in the untreated cells to 38 ng/μg in the cells treated with 30 μM inhibitor **3** or to 36 ng GAGs/μg protein in the cells treated with 80 μM of **11** (Fig. 4B). This result, again, shows a relatively higher potency of inhibitor **3.**

### Increased GAG catabolism in MPS-I fibroblasts treated with the DS-epi1 inhibitors

**Fig. 5:**
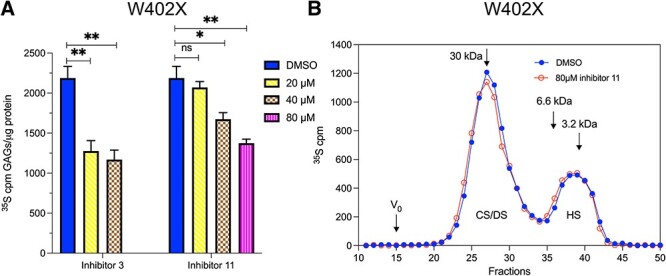
Enhanced catabolism of GAGs in MPS-I fibroblast treated with the inhibitors. MPS-I W402X cells were cultured in the presence of ^35^S-sulfate for 3 days (without inhibitors) to label the GAGs. Then the cells were washed and transferred into fresh medium without ^35^S-sulfate, but in the presence of the inhibitors and cultured for 4 days (chasing). The media, either with **3** and **11** or with DMSO as control was refreshed every second day. A) The remaining labeled GAGs in the cells with and without inhibitors. B) Size of the GAG chains analyzed on Superose 6 column. The experiments were repeated twice with similar results, and the analytical procedure were in triplicates.

After seeing that the inhibitors were able to reduce the overall amount of IdoA and GAGs, we wanted to know whether the effect is only contributed by reduction of biosynthesis. To check the catabolism of GAGs, we applied the pulse-chase experiment. W402X cells were cultured in the presence of ^35^S-sulfate but without the inhibitors for 3 days till confluency to metabolically label the GAGs, then divided into 24 wells and cultured for another 4 days with fresh medium without ^35^S-sulfate (chasing period) in the presence of the inhibitors. Quantification of cellular ^35^S-labeled GAGs shows that the inhibitors, in a concentration-dependent manner, increased the catabolism of the neo-synthesized GAGs (Fig. 5A). Inhibitor **3** treatment reduced the GAG content by 47% at the concentration of 40 μM and inhibitor **11** reduced by 24% at the concentration of 40 μM and 37% at the concentration of 80 μM, when compared to untreated cells.

To check the chain distribution, the GAGs isolated from W402X MPS-I after chasing for 4 days were analyzed by size exclusion chromatography on Superose 6 column. The results show no difference in the chain distribution of both CS/DS and HS between the two samples with or without treatment with the inhibitor **11** at 80 μM (Fig. 5B). The chromatograms of GAGs isolated from cells treated with 20 and 40 μM of **11** or 20 or 40 μM of **3** were superimposable with the profiles presented (data not shown), indicating that the inhibitors did not affect chain fragmentation. The identity/purity of CS/DS and HS were confirmed by chondroitinase ABC treatment and nitrous acid cleavage at pH 1.5, respectively (Supplementary Fig. 1).

### Specificity of the inhibitors

**Fig. 6:**
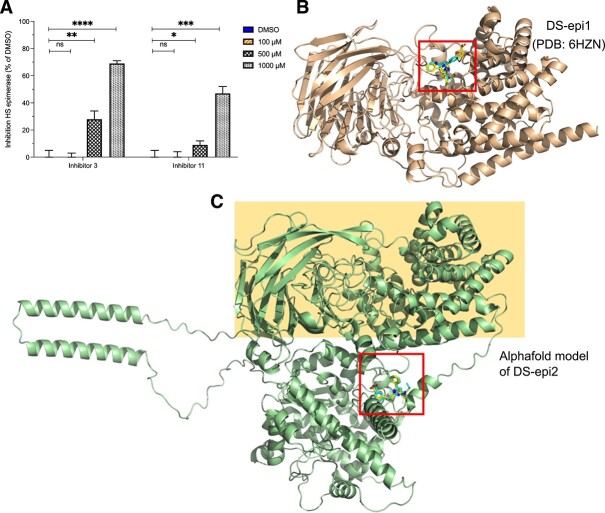
Specificity of inhibitors **3** and **11**. A) Inhibition curves in HSepi enzymatic assay. Each single point represents value of triplicates and the experiment has been repeated two times. Comparison of crystal structure of DS-epi1 (B) and the Alphafold model of DS-epi2 (C) shows the similarity of the epimerase domains of DS-epi1 with DS-epi2 (*highlighted*). Blind docking shows that inhibitors **3** and **11** bind to the epimerase domain of DS-epi1but to the putative sulfotransferase domain of DS-epi2 (*squares*), and therefore are unlikely to modify the DS-epi2 epimerase activity.

To find out whether the inhibitors are specific towards DS-epi1, we tested their activity towards the functionally related enzymes HSepi (involved in HS synthesis) and DS-epi2 (involved in CS/DS synthesis), as these enzymes have a similar substrate recognition property to catalyze epimerization of glucuronic acid to iduronic acid at polymer level (Valla et al. 2001) (Pacheco et al. 2009b). Neither of the two inhibitors displayed HSepi inhibitory activity at the concentration of 100 μM, but both showed some inhibitory activity at higher concentrations. Inhibitor **3** inhibited 28 and 69% HSepi activity at 500 and 1,000 μM, respectively, and **11** inhibited 9 and 47% at 500 and 1,000 μM, respectively (Fig. 6A).

Unfortunately, we were unable to test the inhibitory activity towards DS-epi2, due to technical problems in producing recombinant enzymes. Alternatively, we evaluated the potential of the inhibitors in interaction with DS-epi2 by computer-aided study. DS-epi1 consists of an N-terminal 22–690 aa. epimerase domain and a C-terminal 691–958 aa domain with unknown function, while DS-epi2 has a 27–720 aa. epimerase domain, followed by a C-terminal 824–1,222 domain which has similarity with CS/DS O-sulfotransferases (Pacheco et al. 2009b). However, the actual enzymatic activity of the -sulfotransferase domain has never been proved. Comparing the DS-epi1 molecular structure with the AlphaFold structure of DS-epi2 confirms the similarity of the epimerase domains of DS-epi1 and DS-epi2 (Fig. 6B and C) (Hasan et al. 2021). Docking study using the AlphaFold structure of DS-epi2 revealed that both inhibitors bind to the protein, but to the putative sulfotransferase domain, which is distant from the epimerase active site.

## Discussion

Encouraged by our previous finding that ebselen was able to inhibit DS-epi, thereby reducing IdoA biosynthesis and GAG accumulation in MPS-I cells, we continued the study by looking for novel compounds that can specifically inhibit DS-epi1.

First, DS-epi1 as a pharmacological target was validated by its deletion by CRISPR-Cas9 in fibroblasts cells from patients and normal individuals as control. We choose fibroblasts as cell model not only because they are readily available, but most importantly because CS/DS accumulates 3 times more than HS in the MPS-I fibroblasts (Maccarana et al. 2021). Therefore, a reduction of IdoA in CS/DS is expected to have higher impact than reduction of IdoA in HS for reducing GAG accumulation in these cells. Elimination of DS-epi1 in both WT and MPS-I fibroblasts led to reduced IdoA production in CS/DS and reduction in total GAGs accumulation, confirming the validity of the substrate reduction approach.

Based on our previous findings, the rationale is to look for small molecular compounds that specifically inhibits DS-epi1. Since the molecular structure of DS-epi1 was not available when the project was initiated, chondroitinase AC was used as model for the virtual screening of potential inhibitors. Although chondroitinase AC is a lyase and catalyzes different reaction from DS-epi1, both enzymes bind to same substrate of CS/DS and act on glucuronic acid. Further, the same critical amino acids had superimposable positions in their catalytic sites (Pacheco et al. 2009a). Indeed, from the library established by virtual screening, 17 compounds were detected having high inhibitory activity for DS-epi1 in the epimerase activity assay using purified recombinant DS-epi1. However, limited by the availability of chemical quantity in the library, we were able to select two compounds belonging to different chemical classes for resynthesis andfor further studies. Both of the inhibitors **3** and **11** reduced IdoA production in CS/DS and cellular GAG accumulation in WT and MPS-I cells, in a concentration dependent manner**.** These are the first hits showing high specificity for DS-epi1 with an IC_50_ of approximately 10 μM, though HSepi was partially inhibited at a concentration approximately 100 times higher than the IC_50_ for DS-epi1. The specificity of the inhibitors was further supported by docking studies showing that the inhibitors **3** and **11** bound to DS-epi1 in the catalytic site of the epimerase domain but not to the same domain in DS-epi2. Thus, it is unlikely that the inhibitors would inhibit the epimerase activity of DS-epi2, which should be of interesting to clarify in future studies.

Through the radioisotope incorporation pulse-chase experiment, we found that the novel inhibitors also promoted catabolism of the GAGs in MPS-I cells, a similar effect as ebselen (Maccarana et al. 2021). It is worth noting that although the total radio-labeled GAGs were substantially reduced after chasing for 4 days in the MPS-I cell treated with the inhibitors compared to cells similarly chased in the absence of inhibitors, the overall chain length of the remaining GAGs was the same between the treated and non-treated cells. The accumulated CS/DS in both treated and untreated MPS-I cells appeared as full-length chains (approx. 30 kDa), which may suggest that the catabolism of CS/DS in MPS-I cells is mainly through exo-enzyme degradation instead of internal fragmentation, putatively by hyaluronidase. However, the average chain length of HS in both treated and untreated MPS-I cells is substantially shorter (approx. 4 kDa), suggesting an initial fragmentation by heparanase regardless of presence or absence of the inhibitors. It is still debated about the molecular catabolic mechanism(s) involved in the effect of substrate reduction for treatment of MPSs (Banecka-Majkutewicz et al. 2012). Concerning our findings, one hypothesis is that reduction of the “stop” (IdoA) in the chains may facilitate the action of other degradation enzymes. Nevertheless, since both types of the inhibitors (ebselen and the current compounds) displayed similar phenotypes, suggesting that there should be a common mechanism yet unknown.

Substrate reduction therapy (SRT) is in clinical use for other lysosomal storage disorders (Coutinho et al. 2016) and has been validated for treatment of mucopolysaccharidosis (MPS-IIIa) by reduction of HS biosynthesis in mouse models (Lamanna et al. 2012). One promising result is that using isoflavone genistein reduced GAG synthesis in mice, leading to a clinical trial to treat MPS IIIA and MPS IIIB patients. Despite urinary GAGs was decreased in treated patients, no clinical improvement was noted (de Ruijter et al. 2012). The activity of this compound is via an effect on EGF-mediated signaling pathway, which may have affected other biological activities. Regardless the report that use of rhodamine B, a non-specific inhibitor of GAG biosynthesis, produced limited benefit on MPS-I mice (Derrick-Roberts et al. 2017), our accumulated information from DS-epi1 knockout mice and MPS-I fibroblast cells support the rationale to ameliorate MPS-I by a SRT approach through partial inhibition of DS-epi1.

In summary, aimed at looking for DS-epi1 inhibitors as potential SRT therapy for MPS-I, we, for the first time, report the identification of two small molecular compounds that displayed specific inhibitory activity towards DS-epi1through binding to the catalytic site. These novel inhibitors reduced IdoA formation in CS/DS and accumulation of GAGs in MPS-I cells. The effect and low toxicity of the inhibitors allow further elaboration to develop drug lead compounds.

## Material and methods

### Reagents and analytical tools

^35^S-sodium sulfate (1,500 Ci/mmol) was from PerkinElmer. Sulfate-free Dulbecco’s modified Eagle’s medium (DMEM) (AS31600 cat no. 074-91083P) was from Gibco. Superose 6 10/300, Superdex Peptide 10/300, PD-10 columns, Sephadex G-25, DEAE-Sephacel were from Cytiva. DS-epi1 was detected by Western blot with 1 μg/mL of an immunopurified anti-DS-epi1 rabbit polyclonal antibody obtained by immunization with the peptide antigen (KWSKYKHDLAAS, corresponding to amino acids 509 to 520 of the human/murine sequence; Innovagen, Sweden) (Maccarana et al. 2006). The Biorad stain-free gels were used, and both loading control, i.e. the blotted bands, and the chemioluminiscent signal were visualized with the ChemiDoc Imaging System (Biorad). Cell number was evaluated by DNA staining with crystal violet. Viability was assessed with PrestoBlue™ Cell Viability Reagent (Invitrogen).

### Synthesis inhibitors 3 and 11

Inhibitors 3 and 11 were synthesized by GlycoNovo (Shanghai, China) using routine methods as describled in the supplemental data and solved in DMSO at the concentration of 50 mM.

### Cell cultures

Primary human skin fibroblasts, derived from 1-year-old MPS-I patients and a healthy age-matched donor, were obtained from the “Cell line and DNA Biobank from Patients affected by Genetic Diseases” (Institute G. Gaslini, Genova, Italy). Two MPS-I fibroblasts from two patients were analyzed. One patient had a homozygous stop codon in the iduronidase (*IDUA*) gene, coding for the truncated protein W402X, which is the most common MPS-I variants (31% worldwide) (Kubaski et al. 2020). The second patient had the compound heterozygote *IDUA* mutation coding for P533R/G51D. Analysis of the fibroblast lysates using the fluorogenic substrate 4-methylumbelliferyl-α-L-iduronide (Ou et al. 2014) confirmed extremely reduced iduronidase activity in these fibroblasts (5,000 times less than WT fibroblasts). The cells were maintained in DMEM, 10% FBS (2% FBS in experiments where inhibitors were used) and 100 units/mL penicillin and 100 μg/mL streptomycin.

### CRISPR-Cas 9 mediated DS-epi1 inactivation

Disruption of the DS-epi1 gene was performed according to the method as described before (Joung et al. 2017). Briefly, sgRNA caccgCATTGCAGCCCGCCTCACGG containing the DS-epi1 specific guide sequence (underlined) was inserted into the LentiCRISPRv2 puro plasmid (Addgene cat. no. 98290). The target plasmid, together with the envelope plasmid pMD2.G (Addgene cat. no. 12259) and the packaging plasmid psPAX2 (Addgene, cat. no. 12260) were transfected into Lenti-X 293 cells. The resulting viral particles (either mock or containing the DS-epi1 guide sequence) were used to infect the fibroblasts. Cells were subsequently selected by puromycin resistance.

### Isolation of labeled GAGs and proteoglycans from medium

The cells were plated in complete medium and changed to sulfate-free DMEM, 2% FBS, 10 units/mL penicillin and 10 μg/mL streptomycin the day after (at confluency of 90%). For metabolic labeling, 100 μCi/mL^35^S-sulfate was added in the culture. After 24 h, the medium was recovered for isolation of GAGs or proteoglycans (PGs). The metabolically labelled total PGs were isolated by DEAE-Sephadex anion-exchange chromatography. The eluted PGs were treated with nitrous acid at pH 1.5 to depolymerize HS, and the resultant CS/DS PGs were applied to a Superose 6 column to collect decorin/biglycan. After desalting, the CS/DS chains were released from the core protein by alkaline β-elimination (0.5 M NaOH 4 °C, 0.1 M sodium borohydrate for 16 h). Finally, CS/DS chains were recovered after gel chromatography on a Superose 6 column (the above preparation is valid for Fig. 1B). Alternatively (Fig. 4A), the total labeled GAGs in medium were released from core proteins by alkaline β-elimination and the free GAGs (both HS and DS/CS) were isolated by DEAE-Sephadex chromatography. HS was depolymerized by nitrous acid pH 1.5 and CS/DS chains were recovered after size separation on a Superose 6 column. The purity of CS/DS was verified by analytical digestion with chondroitinase ABC (Sigma C3667) that depolymerized completely the ^35^S-labeled chains.

### Analysis of IdoA in CS/DS chain

IdoA in radiolabeled CS/DS was quantified after degradation by chondroitinase B that only cleaves the *N*-acetyl galactosamine~iduronic acid linkages. Incubations were in 50 μL of 20 mM Hepes, pH 7.2, 50 mM NaCl, 4 mM CaCl_2_, 0.1 mg/mL BSA and 2 mIU chondroitinase B (R&D System). After 2 h at 37 °C the products were separated on a Superdex Peptide column, radioactivity was measured by scintillation counting, and the percentage of iduronic acid over the total iduronic acid + glucuronic acid was calculated.

### Isolation and analysis of unlabeled GAGs from cells

Mock and DS-epi1 depleted fibroblasts were cultured in 75 cm^2^ flasks till 100% confluency (Fig. 1C). To measure the effects of the inhibitors on the cellular GAGs (Fig. 4B), fibroblasts were cultured in 75 cm^2^ flasks till 10% confluency and treated with inhibitor **3** or **11** or DMSO as control. The media were changed every second day with fresh media containing inhibitors for a total of 10 days of cultivation. In both experiments, the cells were trypsinized, washed with PBS and the cell pellet was lysed in 20 mM MES pH 6.5, 150 mM NaCl and 0.1% Triton. After centrifugation, the supernatant was analyzed for protein concentration by the Bradford assay (Bio-Rad), and the GAGs were purified by pronase digestion and DEAE-Sephacel chromatography and quantified by carbazole reaction (Bitter and Muir 1962).

### Pulse-chase labeling GAGs with 35S-sulfate and analysis

MPS-I fibroblasts in a 75 cm^2^ flask were cultured in sulfate-free DMEM, 10% FBS, 10 units/mL penicillin and 10 μg/mL streptomycin with addition of ^35^S-sulfate (100 μCi/mL) for 72 h. Then cells were sub-cultured into 12-well plates and changed to complete DMEM unlabeled medium, i.e. without ^35^S-sulfate, containing 2% FBS and variable concentrations of inhibitors. The medium was refreshed after 2 days. After 4 days of chasing, the cells were trypsinized, washed with PBS and lysed in 100 μL 20 mM MES pH 6.5, 150 mM NaCl, 0.1% Triton. Proteins were quantified and the ^35^S-labeled GAGs were purified by DEAE anion exchange chromatography and quantified. An aliquot of the purified ^35^S-labeled GAGs was released from the core protein by beta-elimination, and applied to Superose 6 column eluted with 0.2 M ammonium bicarbonate at 0.25 mL/min. The eluted fractions were collected every 2 min for radioactivity quantification. GAGs of known size were used as molecular weight markers as indicated in the chromatograms (Petersen et al. 1999).

### Assay of DS-epi1 and HS epimerase

To screen the compound library, recombinant DS-epi1 was purified from the supernatant of HEK293 cells that were transiently transfected with the full-size (amino acid 1–958) human DS-epi1-myc-HIS sequence inserted in the pcDNA.3 expression vector (Maccarana et al. 2006). For the studies with selected inhibitor **3** and **11**, the human DS-epi1used was the truncated form (amino acid 23–775-HIS) as described (Tykesson et al. 2016). The activity of DS-epi1 was assayed as described before (Maccarana et al. 2021)**.**

Human heparan sulfate glucuronyl C-5 epimerase (GLCE, UniProtKB 094923) cDNA sequence corresponding to amino acids 29–617 was inserted into the expression plasmid pLVX-puro (Clontech, USA) and 293HEK cells were transfected by Lipofectamine 3000 reagent (Invitrogen). Stable bulk pool was selected by puromycin at 2.5 ug/ml. The resistant pool of the transfected cells was lysed and epimerase activity was analyzed with and without the inhibitors by the method described in (Maccarana et al. 2021).

### Molecular docking

Interaction of inhibitors **3** and **11** with the molecular structure of DS-epi1 (PDB ID: 6HZN) and Alphafold model of DS-epi-2 was studied by molecular docking utilizing Autodock VINA in YASARA (Wong et al. 2005; Trott and Olson 2010). To remove bumps and ascertain the covalent geometry of the ligands, the structures were all energy-minimized via the NOVA force field (Krieger et al. 2002). The blind dockings were undertaken by defining simulation cell boxes of 100.00 Å × 100.00 Å × 100.00 Å for DSepi-1, and 130.00 Å × 130.00 Å × 130.00 Å for DS-epi2 Alphafold model. The docking studies were carried out through the built-in docking simulation macro “dock_run.mcr” using AMBER03 force field (Duan et al. 2003).

### Statistical analysis

Statistical significance was analyzed by the 2-tailed unpaired Student’s t-test. Values of *P* < 0.05 were considered significant. ^*^ means *P* < 0.05, ^**^ means *P* < 0.01, ^***^ means *P* < 0.001, ^****^P < 0.0001.

## Supplementary Material

fig_S1-v2_cwae025

Supplementary_Table_I_cwae025

Supplementary_Table_II_cwae025

20231212_Supplemental_data_cwae025

## Data Availability

The data underlying this article are available in the article and in its online supplementary material.

## CRediT author statement

Marco Maccarana (Conceptualization [equal], Data curation [equal], Formal analysis [equal], Investigation [equal], Methodology [equal], Validation [equal], Visualization [equal], Writing—original draft [equal], Writing—review & editing [equal]), Binjie Li (Data curation [supporting], Investigation [supporting], Methodology [supporting], Writing—original draft [supporting], Writing—review & editing [supporting]), Honglian Li (Data curation [supporting], Formal analysis [supporting], Writing—review & editing [supporting]), Jianping Fang (Data curation [supporting], Formal analysis [supporting], Investigation [supporting], Methodology [supporting], Resources [supporting], Writing—review & editing [supporting]), Mingjia Yu (Data curation [supporting], Formal analysis [supporting], Methodology [supporting], Writing—review & editing [supporting]), and Jin-ping Li (Conceptualization [equal], Data curation [equal], Formal analysis [equal], Funding acquisition [lead], Investigation [equal], Methodology [equal], Project administration [lead], Resources [lead], Visualization [equal], Writing—original draft [equal], Writing—review & editing [equal]).
