# Supplementary material for: Inhibitors of dermatan sulfate epimerase 1 decreased accumulation of glycosaminoglycans in mucopolysaccharidosis type I fibroblasts
Source: Glycobiology. 2024 May 17;34(6):cwae025. doi: 10.1093/glycob/cwae025 (PMC11101759; doi:10.1093/glycob/cwae025)
Supplement: Supplementary_Table_I_cwae025 [file supplementary_table_i_cwae025.docx]

**Supplementary Table I.** In silico binding affinity of inhibitor **3** and **11** with human dermatan sulfate epimerase 1 (PDB ID: 6HZN).

| Ligand name | Binding Free Energies (−kcal/mol) | Dissociation Constant (μM) |
| --- | --- | --- |
| Inhibitor **11** | 7.484 | 3.267 |
| Inhibitor **3** | 8.352 | 0.755 |
