# Supplementary material for: Inhibitors of dermatan sulfate epimerase 1 decreased accumulation of glycosaminoglycans in mucopolysaccharidosis type I fibroblasts
Source: Glycobiology. 2024 May 17;34(6):cwae025. doi: 10.1093/glycob/cwae025 (PMC11101759; doi:10.1093/glycob/cwae025)
Supplement: Supplementary_Table_II_cwae025 [file supplementary_table_ii_cwae025.docx]

**Supplementary Table II.** In silico binding affinity of inhibitor **3** and **11** with Chondroitinase AC (PDB ID: 1HMW).

| Ligand name | Binding Free Energies (−kcal/mol) | Dissociation Constant (μM) |
| --- | --- | --- |
| Inhibitor **11** | 7.588 | 2.741 |
| Inhibitor **3** | 8.480 | 0.608 |
