# Supplementary material for: Inhibitors of dermatan sulfate epimerase 1 decreased accumulation of glycosaminoglycans in mucopolysaccharidosis type I fibroblasts
Source: Glycobiology. 2024 May 17;34(6):cwae025. doi: 10.1093/glycob/cwae025 (PMC11101759; doi:10.1093/glycob/cwae025)
Supplement: 20231212_Supplemental_data_cwae025 [file 20231212_supplemental_data_cwae025.docx]

**Supplemental methods**

*Synthesis of inhibitors* ***3*** *and* ***11***

**Example 1**

**3-(((4-(methyl(propyl)amino)-6-phenylpyrimidin-2-yl)thio)methyl)benzoic acid**

**Step 1: 6-phenyl-2-thioxo-2,3-dihydropyrimidin-4(1H)-one**

To a solution of benzoylacetate (6.3 g, 32.8 mmol) in EtOH (10 mL) was added NaOEt (20% in EtOH, 10 mL). The mixture was stirred at room temperature for 20 minutes and thiourea (3 g, 39.5 mmol) was then added. The mixture was heated at reflux for 24 hours. After cooling, the solvent was evaporated under reduced pressure and H_2_O (20 mL) was added to the residue. The solution was acidified with HCl 37% (pH = 2). The precipitate was collected by filtration and washed with water to give desired product (1.6 g, 24% yield) as a pale yellow solid.

**Step 2: methyl 3-(((6-oxo-4-phenyl-1,6-dihydropyrimidin-2-yl)thio)methyl)benzoate**

To a stirred solution of 6-phenyl-2-thioxo-2,3-dihydropyrimidin-4(1H)-one (600 mg, 2.94 mmol) and methyl 3-(bromomethyl)benzoate (805 mg, 3.53 mmol) in EtOH(6.0 ml),was added Potassium carbonate (812 mg, 5.88 mmol). The resulting mixture was stirred at room temperature for 2 hours. The crude mixture was evaporated under reduced pressure and H_2_O (20 mL) was added to the residue. The mixture was extracted with EtOAc (15 mLx2), dried over anhydrous Na_2_SO_4_ and evaporated under reduced pressure to give the desired product (285 mg, 27.5% yield) as a yellow solid.

**Step 3:** **methyl 3-(((4-chloro-6-phenylpyrimidin-2-yl)thio)methyl)benzoate**

To a solution of methyl 3-(((6-oxo-4-phenyl-1,6-dihydropyrimidin-2-yl)thio)methyl)benzoate (285 mg, 0.8 mmol) in MeCN (5.0 ml) , was carefully added Phosphorus oxychloride (186 mg, 1.2 mmol) dropwise. The resulting mixture was stirred at 80^o^C for 3 hrs. After cooled down, the crude mixture was evaporated under reduced pressure. The reaction mixture was quenched by the addition of the saturated sodium bicarbonate solution. The mixture was extracted with EtOAc (15 mLx2), dried over anhydrous Na_2_SO_4_ and evaporated under reduced pressure. The residue was purified by Flash chromatography with (16.7%EtOAc,83.3% PE) to give the desired product (300 mg, 100% yield) as a colorless oily substance.

**Step 4: methyl 3-(((4-(methyl(propyl)amino)-6-phenylpyrimidin-2-yl)thio)methyl)benzoate**

To a solution of methyl 3-(((4-chloro-6-phenylpyrimidin-2-yl)thio)methyl)benzoate (250 mg,0.68 mmol) and N-methylpropan-1-amine (74 mg , 1.01 mmol) in MeCN (5.0 ml) , was added DIEA (261 mg, 2.04 mmol). The resulting mixture was stirred at 80^o^C for 3 hrs. After cooled down, the crude mixture was evaporated under reduced pressure. The residue was purified by Flash chromatography with (16.7% EtOAc,83.3% PE) to give the desired product (200 mg,72% yield) as a colorless oily substance. **Step 5: 3-(((4-(methyl(propyl)amino)-6-phenylpyrimidin-2-yl)thio)methyl)benzoic acid**

To a solution of methyl 3-(((4-(methyl(propyl)amino)-6-phenylpyrimidin-2-yl)thio)methyl)benzoate (200 mg, 0.491 mmol ) in THF (1.0 mL) and MeOH(1.0 mL) was added NaOH (1 mL, 2.47 mmol) and the reaction mixture was stirred at room temperature for 2 hr. The MeOH was evaporated under reduced pressure and the residue was extracted with EtOAc (15 mL) once. The water layer was adjusted with 1N HCl solution until pH~4 and extracted with EtOAc (15 mL x 2). The organic layers were dried over anhydrous Na_2_SO_4_ and concentrated under reduced pressure. The residue was purified by Flash chromatography with (16.7% EtOAc,83.3% PE) to give the desired product (110 mg,57% yield) as a white solid.

1H NMR (400 MHz, DMSO-d6) δ 8.10 – 7.93 (m, 3H), 7.78 (d, J = 7.6 Hz, 1H), 7.67 (d, J = 7.6 Hz, 1H), 7.56 – 7.38 (m, 4H), 6.83 (s, 1H), 4.48 (s, 2H),3.45-3.40(m,2H) 3.10 (s, 3H), 1.61 – 1.42 (m, 2H), 0.90 – 0.70 (m, 3H). LC-MS (ESI) m/z [M+H]^+^394.0.

**Example 1**

**5-(((4-(cyclohexyl(methyl)amino)-6-phenylpyrimidin-2-yl)thio)methyl)furan-2 carboxylic acid**

**Step 1: methyl 5-(bromomethyl)furan-2-carboxylate**

To a solution of methyl 5-methylfuran-2-carboxylate (1.0 g, 7.14 mmol) and NBS (1.53 g, 8.57 mmol) in Carbon tetrachloride (10 mL) was added BPO (173 mg, 0.71 mmol). The mixture was stirred at 80^o^C for 3 hrs. After cooled down, the crude mixture was evaporated under reduced pressure. The residue was purified by Flash chromatography with (16.7% EtOAc, 83.3% PE) to give the desired product (1.5 g, 96.3% yield) as a colorless oily substance.

**Step 2: methyl 5-(((6-oxo-4-phenyl-1,6-dihydropyrimidin-2-yl)thio)methyl)furan-2-carboxylate**

To a stirred solution of methyl 5-(bromomethyl)furan-2-carboxylate (750 mg, 3.44 mmol) and 6-phenyl-2-thioxo-2,3-dihydropyrimidin-4(1H)-one (585 mg, 2.87 mmol) in EtOH(7.0 ml),was added Potassium carbonate (792 mg, 5.74 mmol). The resulting mixture was stirred at 80^o^C for 3 hrs. After cooled down, the crude mixture was evaporated under reduced pressure. The residue was purified by Flash chromatography with (75% EtOAc, 25% PE) to give the desired product (280 mg, 23.8% yield) as a yellow solid.

**Step 3:** **methyl 5-(((4-chloro-6-phenylpyrimidin-2-yl)thio)methyl)furan-2-carboxylate**

To a solution of methyl 5-(((6-oxo-4-phenyl-1,6-dihydropyrimidin-2-yl)thio)methyl)furan-2-carboxylate (280 mg, 0.82 mmol) in MeCN (5.0 ml) , was carefully added Phosphorus oxychloride (188 mg, 1.23 mmol) dropwise. The resulting mixture was stirred at 80^o^C for 3 hrs. After cooled down, the crude mixture was evaporated under reduced pressure. The reaction mixture was quenched by the addition of the saturated sodium bicarbonate solution. The mixture was extracted with EtOAc (15 mLx2), dried over anhydrous Na_2_SO_4_ and evaporated under reduced pressure. The residue was purified by Flash chromatography with (16.7% EtOAc, 83.3% PE) to give the desired product (290 mg,98.3% yield) as a red oily substance.

**Step 4:** **methyl 5-(((4-(cyclohexyl(methyl)amino)-6-phenylpyrimidin-2-yl)thio)methyl)furan-2-carboxylate**

To a solution of methyl 5-(((4-chloro-6-phenylpyrimidin-2-yl)thio)methyl)furan-2-carboxylate (270 mg,0.75 mmol) and cyclohexylamine (127 mg , 1.13 mmol) in MeCN (5.0 ml) , was added DIEA (290 mg, 2.25 mmol). The resulting mixture was stirred at 80^o^C for 3 hrs. After cooled down, the crude mixture was evaporated under reduced pressure. The residue was purified by Flash chromatography with (16.7 EtOAc,83.3% PE) to give the desired product (170 mg, 51.9% yield) as a yellow oily substance. **Step 5: 5-(((4-(cyclohexyl(methyl)amino)-6-phenylpyrimidin-2-yl)thio)methyl)furan-2-carboxylic acid**

To a solution of methyl methyl 5-(((4-(cyclohexyl(methyl)amino)-6-phenylpyrimidin-2-yl)thio)methyl)furan-2-carboxylate (170 mg, 0.40 mmol ) in THF (1.0 mL) and MeOH(1.0 mL) was added NaOH (1 mL, 2.47 mmol) and the reaction mixture was stirred at room temperature for 2 hr. The MeOH was evaporated under reduced pressure and the residue was extracted with EtOAc (15 mL) once. The water layer was adjusted with 1N HCl solution until pH~4 and extracted with EtOAc (15 mL x 2). The organic layers were dried over anhydrous Na_2_SO_4_ and concentrated under reduced pressure. The residue was purified by Flash chromatography with (16.7 EtOAc, 83.3% PE) to give the desired product (95 mg, 57.8% yield) as a white solid.

1H NMR (400 MHz, Chloroform-d) δ 7.95 – 7.91 (m, 2H), 7.41 (d, J = 5.4 Hz, 3H), 7.10 (s, 1H), 6.45 (s, 1H), 6.34 (s, 1H), 4.42 (s, 2H), 2.91 (s, 3H), 1.80 (d, J = 11.9 Hz, 2H), 1.67 (s, 3H), 1.49 – 1.05 (m, 6H). LC-MS (ESI) m/z [M+H]^+^424.0.

**Legend to Supplemental Fig. 1**

The identity/purity of CS/DS and HS were confirmed by chondroitinase ABC treatment and nitrous acid cleavage at pH 1.5, respectively. The GAGs isolated from W402X MPS-I after chasing for 4 days in the presence of 80 µM of inhibitor **11**were either left untreated or treated with nitrous acid, or treated with chondroitinase ABC. The samples were then analyzed by size exclusion chromatography on Superose 6 column. Results show that the prominent peak is entirely constituted by CS/DS, while the minor peak is only HS. Identical results were obtained when GAGs from control cells, i.e. not treated with any inhibitor, or cells treated with 20 or 40 µM inhibitor **11** were analyzed.
